# *Corydalis edulis* Maxim. Promotes Insulin Secretion via the Activation of Protein Kinase Cs (PKCs) in Mice and Pancreatic β Cells

**DOI:** 10.1038/srep40454

**Published:** 2017-01-16

**Authors:** Jiao Zheng, Yunfang Zhao, Qixing Lun, Yuelin Song, Shepo Shi, Xiaopan Gu, Bo Pan, Changhai Qu, Jun Li, Pengfei Tu

**Affiliations:** 1 Modern Research Center for Traditional Chinese Medicine, Beijing University of Chinese Medicine, Beijing 100029, China; 2 School of Chinese Materia Medica, Beijing University of Chinese Medicine, Beijing 100102, China

## Abstract

*Corydalis edulis* Maxim., a widely grown plant in China, had been proposed for the treatment for type 2 diabetes mellitus. In this study, we found that *C. edulis* extract (CE) is protective against diabetes in mice. The treatment of hyperglycemic and hyperlipidemic apolipoprotein E (ApoE)−/− mice with a high dose of CE reduced serum glucose by 28.84% and serum total cholesterol by 17.34% and increased insulin release. We also found that CE significantly enhanced insulin secretion in a glucose-independent manner in hamster pancreatic β cell (HIT-T15). Further investigation revealed that CE stimulated insulin exocytosis by a protein kinase C (PKC)-dependent signaling pathway and that CE selectively activated novel protein kinase Cs (nPKCs) and atypical PKCs (aPKCs) but not conventional PKCs (cPKCs) in HIT-T15 cells. To the best of our knowledge, our study is the first to identify the PKC pathway as a direct target and one of the major mechanisms underlying the antidiabetic effect of CE. Given the good insulinotropic effect of this herbal medicine, CE is a promising agent for the development of new drugs for treating diabetes.

Diabetes is a metabolic disease and is a major cause of morbidity and mortality worldwide. Over 90% of patients diagnosed with type 2 diabetes mellitus (T2DM)^1^,^2^. It is widely accepted that T2DM mainly results from dysfunction of pancreatic β cells secreting and insulin resistance. Impairment of insulin secretion is an important pathogenetic characteristic of T2DM, causing hyperglycemia and diabetic vascular complications, such as nephropathy, retinopathy, and neuropathy^3^,^4^.

Improving of β cell function is a major goal in the clinical management of diabetes. Sulfonylureas have been available for decades as insulin secretagogues. They bond to their corresponding receptors, increase the triggering signal of insulin secretion by acting on K_ATP_ channels, and depolarize the plasma membrane^5^.

Other molecules that produce major amplifying signals in the insulin secretory process are mainly involved in the activation of protein kinases, particularly protein kinase C (PKC)^6^. The PKC family appears to be ubiquitous in mammalian tissues^7^,^8^, and consist of isoforms classified into three groups: Ca^2+^ and diacylglycerol (DAG)-dependent (conventional isoforms, cPKCs: α, βI, βII and γ), DAG-independent (novel isoforms, nPKCs: δ, ε, η and θ), and Ca^2+^- and DAG-independent isoforms (atypical isoforms, aPKCs: ζ, τ/λ and μ). Glucose-dependent regulation of insulin secretion from β cells is associated with the generation of upstream activators of PKCs, such as DAG^9^. In addition, PKCs were reported to be vitally important for modulation of the insulin secretory response by other receptor-operated secretagogues, including carbachol (CCh)^10^, cholecystokinin (CCK)^11^ and phorbol 12-myristate 13-acetate (PMA)^12^.

Although several herbal extracts or compounds have been reported to ameliorate diabetic symptoms in animal models^13^,^14^, the use of natural products for the treatment of diabetes has not been explored in depth and the molecular targets remain unknown. *C. edulis* (Papaveraceae, Corydalis Vent) is a widely grown plant in eastern Asia, and it has been traditionally used as an anti-bacterial and anti-tussive agent for centuries in China. Recently, this herb has been used to treat patients with T2DM in folk in China. However, the mechanisms underlying its antidiabetic action are not understood. In this study, we found that oral administration of *C. edulis* extract (CE) had an insulinotropic action and lipid-lowering properties in high-fat and high-sucrose (HFHS) diet-fed apolipoprotein E (ApoE) −/− mice. *In vitro,* we found that CE could significantly improve insulin secretion in HIT-T15 cells via the activation of PKCs. Taken together, these data suggest that CE has a potential application as a new agent for the treatment of T2DM in humans.

## Results

### CE decreased fast blood glucose (FBG), total cholesterol (TC), and triglyceride (TG) and increased blood insulin in mice

**Figure 1:**
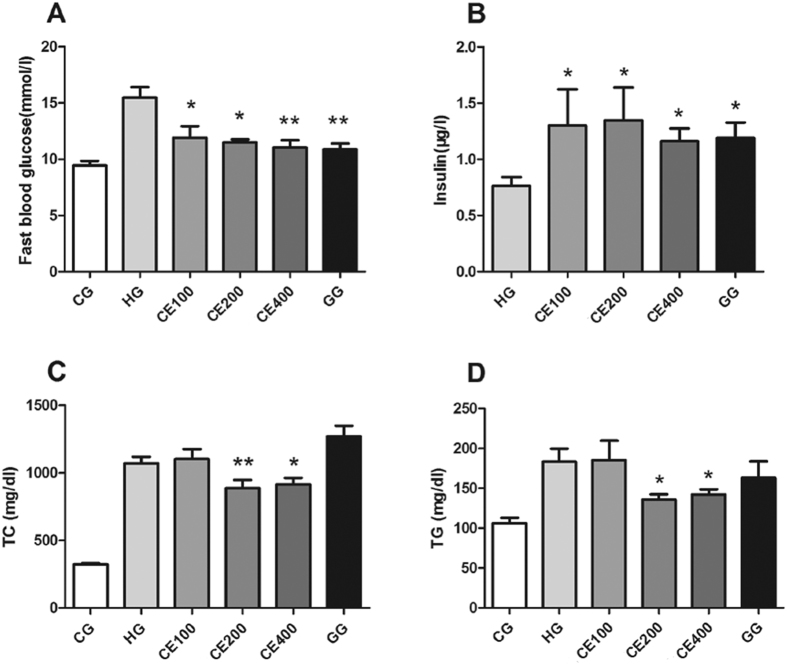
CE decreased fast blood glucose and increased blood insulin levels of ApoE−/− mice fed with HFHS diet. (**A**) Fast blood glucose (FBG) levels of the mice after CE treatment. (**B**) Blood insulin levels of the mice after CE treatment. (**C**) TC levels of the mice after CE treatment. (**D**) TG levels of the mice after CE treatment. *P < 0.05 vs. HG; **P < 0.01 vs. HG, n = 9. CG: chow diet-treated group, HG: HFHS diet-treated group, CE 100: low dose CE-treated group, CE 200: middle dose CE-treated group, CE400: high dose CE-treated group, GG: glicalzide-treated group, TC: total cholesterol, TG: triglycerides.

Because a high-energy diet impaires both glucose tolerance and insulin tolerance, we used the HFHS diet-fed ApoE−/− mice as a model for T2DM and hyperlipidemia. In our study, treatment with the antidiabetic sulfonylurea drug, gliclazide enhanced insulin secretion pancreatic β cells and reduced fasting blood glucose in mice (Fig. A and B). Similar to gliclazide, CE-treatment dose-dependently decreased the glucose levels in the blood (Fig. 1A). In addition, serum TC and TG levels in medium and high dose CE-treated mice were much lower than those of the HFHS diet-treated mice (Figs 1C and D, *P* < 0.05). Blood insulin levels in both CE and gliclazide treatment groups were significantly elevated compared to that of the HG mice (Fig. 1B). These data showed that CE could markedly ameliorate glucose and lipid metabolism and promote insulin release in HFHS-treated ApoE−/− mice. Because CE could control diet-induced metabolic disorders in mice, body weight and food intake were monitored. No significant differences were detected in either body weights or food intakes amount of the groups during the experiment (data not shown).

### CE ameliorated intraperitoneal glucose tolerance test (IPGTT) and intraperitoneal insulin tolerance test (ITT) of the HFHS diet-fed ApoE−/− mice

**Figure 2:**
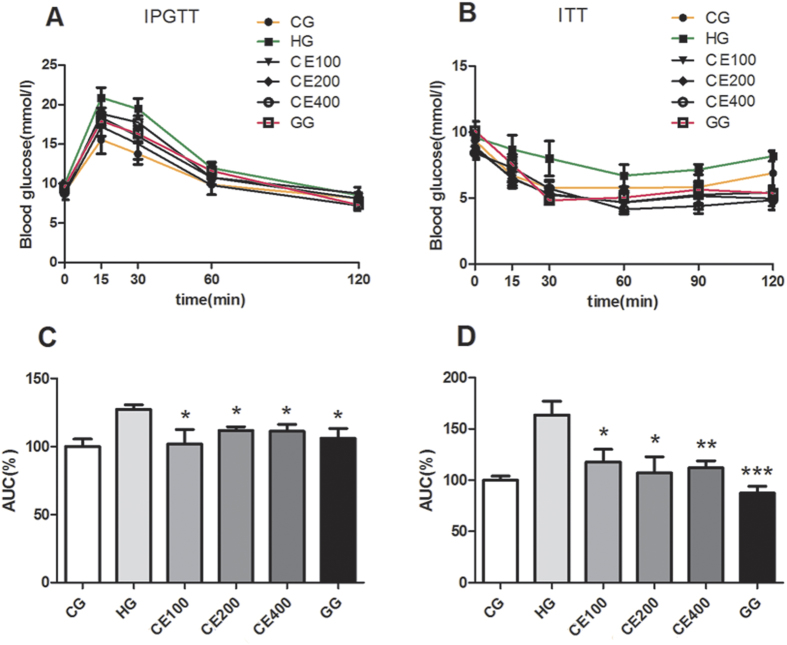
CE improved IPGTT and ITT of ApoE−/− mice fed a HFHS diet. (**A**) Effect of CE on glucose tolerance in HFHS diet-treated mice as determined by intraperitoneal glucose tolerance test (IPGTT). (**B**) Effect of CE on insulin resistance in HFHS diet-treated mice as determined by insulin tolerance test (ITT). (**C**) Quantification of the area under the curve (AUC) from the IPGTT in (**A**). (**D**) Quantification of the AUC of the ITT in (**B**). *P < 0.05 vs. HG; **P < 0.01 vs. HG, ***P < 0.001 vs. HG, n = 9. IPGTT: intraperitoneal glucose tolerance test, ITT: insulin tolerance test, AUC: area under curve.

Because CE decreased glucose and TC levels in plasma, we investigated whether CE could improve glucose and insulin tolerance *in vivo*. HFHS diet-fed mice exhibited impaired glucose and insulin tolerance compared to mice fed chow diet. The results of IPGTT indicated that the CE treatment dose-dependently ameliorated glucose clearance of HFHS diet-fed ApoE−/− mice (Fig. 2A and C). CE treatment significantly decreased peak glucose level 15 min after glucose administration compared to HG at the same time point (*P* < 0.05). Similarly, ITT results revealed that insulin sensitivity in CE-treated mice was increased compared to that in untreated HFHS diet-treated mice. Blood glucose level curves in CE groups were all below that for the HG mice and were even as low as that of the chow diet-treated mice (Fig. 2B and D). Therefore, blood glucose levels were effectively controlled by CE after an intraperitoneal insulin injection, suggesting that CE gradually improved insulin resistance. In addition, impairments in glucose and insulin tolerance in the gliclazide-treated group were ameliorated as compared to the HG mice.

### CE promoted insulin secretion in HIT-T15 cells and stimulated PKC-dependent insulin exocytosis

**Figure 3:**
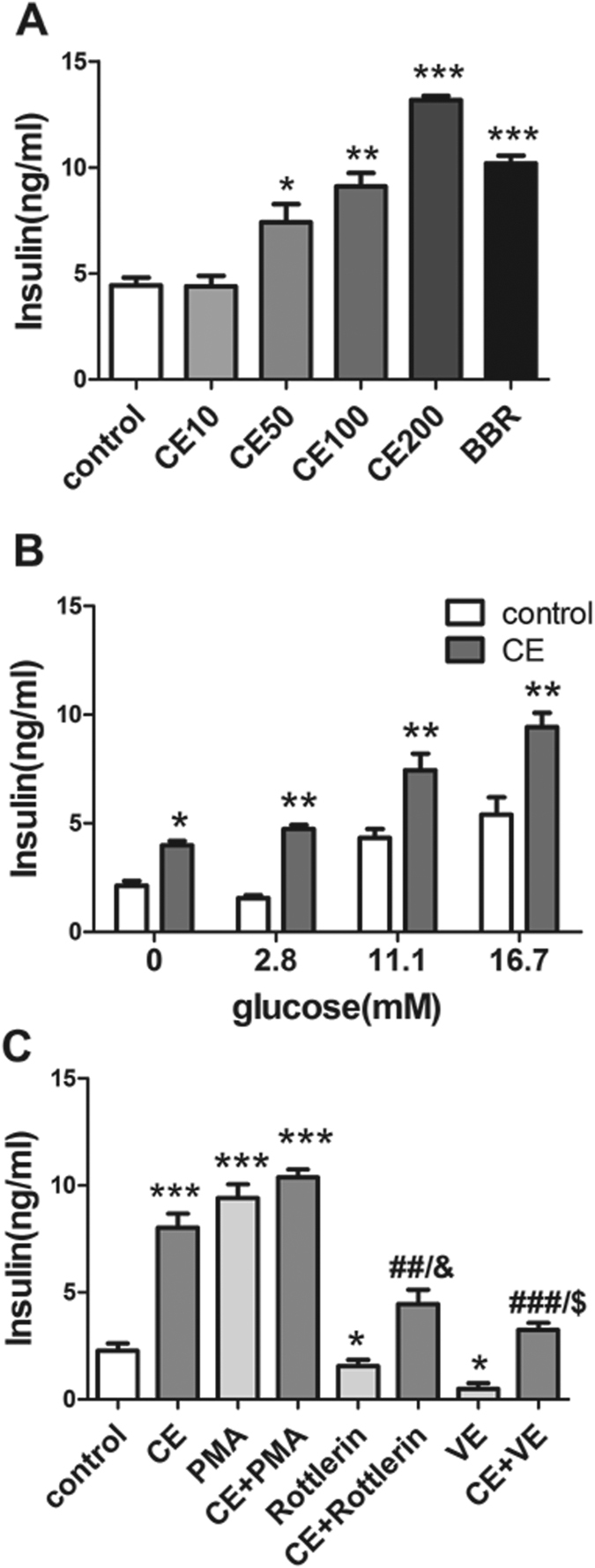
CE enhanced insulin secretion in HIT-T15 cells dose-dependently, glucose-independently, and PKC-dependently. (**A**) HIT-T15 cells were incubated in different concentrations of CE (10, 50, 100, 200 μg/ml) and berberine (50 μM) supplemented with 16.7 mM glucose in KRB for 1 h. (**B**) HIT-T15 cells were switched to medium containing 50 mg/ml CE and increasing concentrations of glucose (0, 2.8, 11.1 and 16.7 mM) for 1 h. (**C**) The insulinotropic effect of CE can be altered by PKC inhibitor rottlerin, DAG inhibitor vitamin E, but the activator PMA. Glucose (16.7 mM)-stimulated insulin secretion from HIT-T15 cells treated with CE (50 μg/ml alone or together with indicated compounds: PMA, 0.1 μM; rottlerin, 5 μM; vitamin E, 200 μM. *P < 0.05 vs. control; **P < 0.01 vs. control; ***P < 0.001 vs. control; ^##^*P* < 0.01 vs. CE; ^&^*P* < 0.05 vs. rottlerin; ^$^*P* < 0.05 vs. vitamin E. Control: DMSO-treated group, CE10: 10 μg/ml CE-treated group, CE50: 50 μg/ml CE-treated group, CE100: 100 μg/ml CE-treated group, CE200: 200 μg/ml CE-treated group, BBR: berberine-treated group, PMA: phorbol 12-myristate 13-acetate, CE+PMA: 50 μg/ml CE- and 0.1 μM PMA-treated group, VE: vitamin E, VE+CE: 50 μg/ml CE and 200 μM VE treated group.

Based on the results *in vivo*, we surmised that CE may be able to promote insulin release. To examine this hypothesis, we established a pancreatic β cell (HIT-T15 cell) model to investigate the effect of CE on insulin secretion. Berberine (BBR) was previously reported to have an insulinotropic effect^15^,^16^, and was used as the positive control in this study. The experiments were performed in the presence of KRB containing 16.7 mM glucose, which is a pathologically relevant condition. Consistent with the results *in vivo*, CE drastically and dose-dependently increased insulin secretion compared to untreated cells (Fig. 3A). Since the metabolism of glucose is an absolute requirement for the stimulation of insulin release, the relationship between the stimulatory action of CE and glucose concentration was analyzed. Cells were treated with CE in the presence of 0, 2.8, 11.1, 16.7 mM glucose, and CE triggered insulin release from 0 to 16.7 mM glucose. Therefore, CE potentiated insulin secretion in a glucose-independent manner (Fig. 3B).

To explore the mechanism of action of the effect of CE on insulin secretion further, PMA, rottlerin, and vitamin E were added to CE-treated HIT-T15 cells in the presence of 16.7 mM glucose (Fig. 3C). Treatment with PMA, a non-selective activator of both cPKCs (α, βI, βII, and γ) and nPKCs (δ, ε, η, and θ), clearly increased insulin secretion, as previously reported^12^. The effect of combining CE and PMA on insulin secretion was not additive. Then we analyzed the effect of CE on insulin secretion in the presence of the PKC δ inhibitor-rottlerin. Consistent with the previously published results^17^, the administration of rottlerin alone remarkably decreased insulin secretion. Although rottlerin significantly inhibited the insulinotropic effect of CE, CE still increased insulin secretion compared with the untreated cells, suggesting that the enhancement of insulin secretion in HIT-T15 cells by CE required not only PKC δ activation but also some other PKC isoforms. Because several studies had shown that vitamin E (VE) can prevent the rise in DAG and subsequent PKC activation in vascular cells exposed to high glucose^18^, we investigated the effect of a DAG inhibitor, VE, on the insulinotropic effect of CE. We found that VE significantly reduced the insulinotropic effect of CE. Taken together, these data suggest that CE potentiated insulin secretion through the activation of DAG and/or PKCs, at least PKC δ. Since the cAMP/protein kinase A (PKA) system is an important enhancer of insulin secretion, the effect on cAMP levels in HIT-T15 cells was tested after CE treatment. The data showed CE did not affect the cAMP levels in HIT-T15 cells (supplementary data, Figure S2).

### CE affected the translocation of PKCs from cytosol to plasma membrane

**Figure 4:**
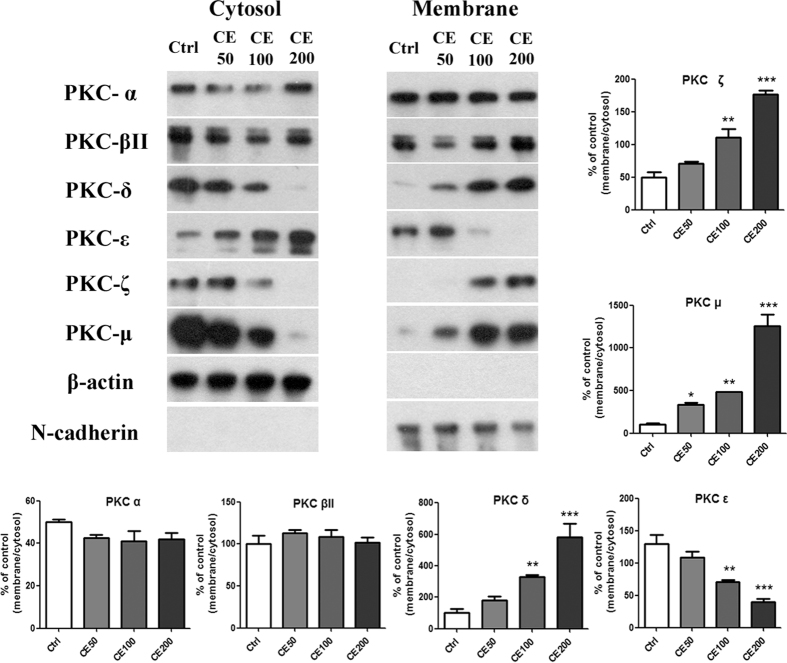
The translocation of PKC isoforms from cytosol to membrane in HIT-T15 cells treated with different concentrations of CE. Cytosolic and membranal fractions were separately loaded on the gels, and the percentage of the control group, corresponding to each PKC’s translocation, were quantified for all PKC isozymes.

cPKC (α, βII), nPKC (δ, ε), and aPKC (ζ, μ) are expressed in HIT-T15 cells^19^. To confirm the involvement of PKCs in the insulinotropic effect of CE, we examined the effect of CE on the activation of PKC isoforms. The translocation of PKCs from cytosol to the cell membrane has frequently been used as a surrogate measure of PKC isoform activation in cells^20^, and we analyzed the translocation patterns of cPKCs (α, βII), nPKCs (δ, ε), and aPKCs (ζ, μ) in HIT-T15 cells with different concentrations of CE (Fig. 4). The translocation of PKC ε was suppressed by CE, whereas those of PKC δ, μ and ζ were promoted. N-cadherin, a membrane marker, is a component of the cytoplasmic membrane and was totally absent from the cytosolic fractions. Similarly, the cytosolic marker β-actin was nearly missing from each membrane fraction.

### Localization of endogenous PKC α, δ, and μ in HIT-T15 cells in response to CE

**Figure 5:**
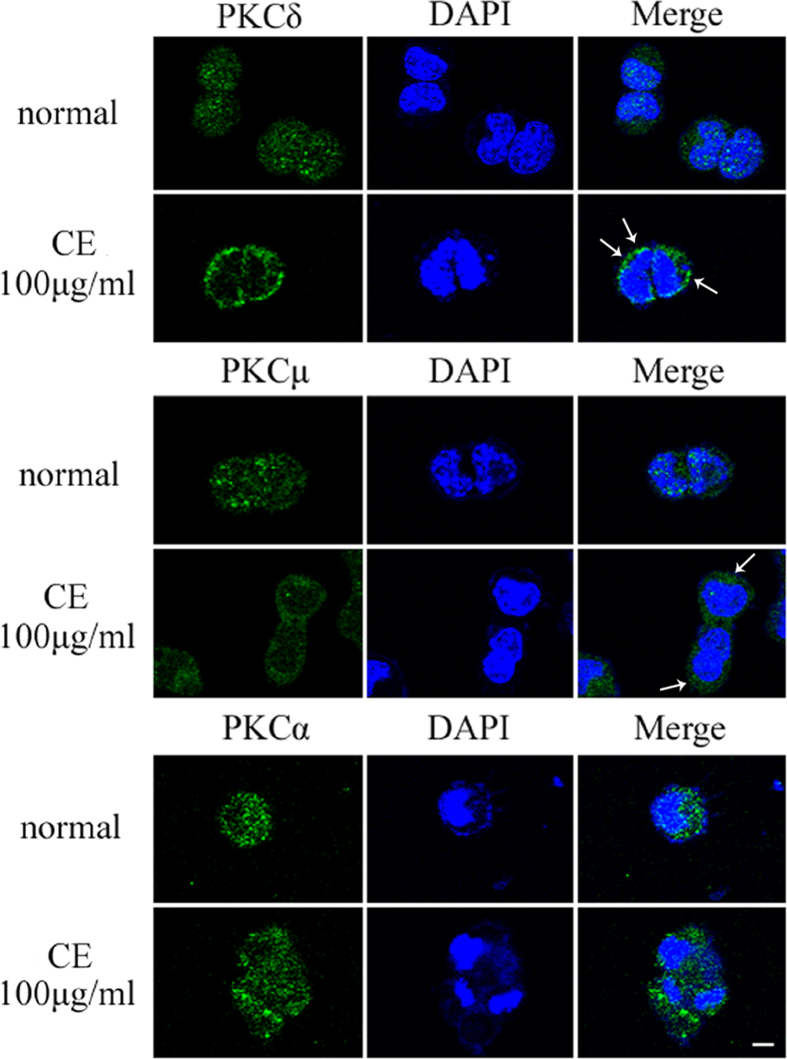
Immunofluorescence staining of PKC α, PKC δ, and PKC μ in HIT-T15 cells. HIT-T15 cells were treated without or with CE (100 μg/ml) for 1 h. The cells were then fixed and stained with various antibodies of PKC isoforms including PKC δ, PKC μ and PKC α and DAPI (a nuclear marker). After treatment, cells were examined by confocal microscopy. Scale bar = 5 μm. Normal: DMSO-treated group, CE100: 100 μg/ml CE-treated group.

Next, we demonstrated the translocation of PKCs in pancreatic β cells by laser confocal microscope. Consistent with the results of Western blotting, the images of immunocytochemistry clearly demonstrated that CE efficiently induced the translocation of endogenous PKC δ and μ from the cytosol to the plasma membrane. PKC α was mainly identified in the cytosol and did not translocate upon treatment with CE (Fig. 5).

### PKC δ, μ, and ζ are required for the insulinotropic effect of CE

**Figure 6:**
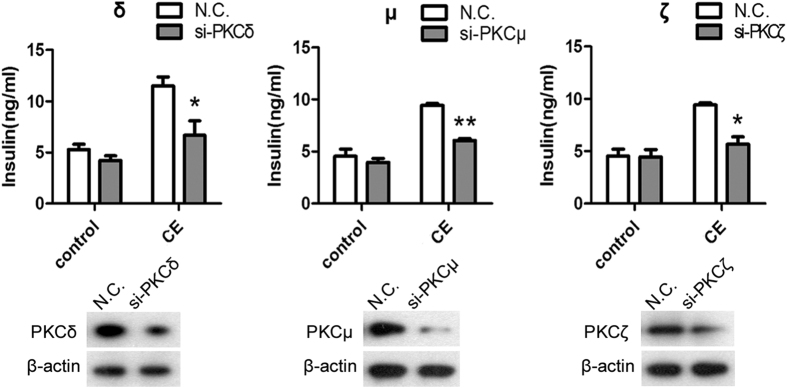
The insulinotropic effects of CE were inhibited in HIT-T15 cells transfected with indicated siRNAs (PKC δ, μ, or ζ). Insulin secretion in PKC δ, μ or ε downregulated cells which were stimulated with DMSO or 100 μg/ml CE. **P < 0.01 vs. N.C. N.C.: negative control siRNA, Control: DMSO-treated group, CE100: 100 μg/ml CE-treated group.

To confirm the involvement of PKC δ, μ, and ζ, we conducted small interfering RNA (siRNA)-mediated loss-of-function experiments with chemically synthesized siRNAs. The results showed that the downregulation of anyone of PKC δ, PKC μ, or PKC ζ by siRNA resulted in a significant inhibition of the insulinotropic effect of CE (Fig. 6). Hence, we speculated that PKC δ, μ, and ζ were required for the insulinotropic role of CE.

## Discussion

Expanding on previous work that reported the conventional efficacy of *C. edulis* in diabetes, we demonstrated for the first time that CE lowered serum glucose and produced beneficial effects in diabetes model mice. CE prevented dietary-induced diabetes in ApoE−/− mice by increasing the insulin secretion. In addition, our data revealed that the insulinotropic effect of CE was mediated by PKC isoforms.

In dietary-induced ApoE−/− mice, all dose of CE tested were effective at controlling blood glucose levels compared to that of HFHS diet-fed mice. Impaired glucose tolerance was markedly improved by CE treatment, and insulin sensitivity was restored to normal levels, as assessed by the IPGTT and ITT, indicating that CE exerted a long-term effect on blood glucose regulation via improvement of insulin resistance. Furthermore, insulin measurements of mice showed that CE had an insulinotropic effect. Taken together, these data indicated that the alleviation of blood glucose by CE was probably attributed to potentiating increases in insulin secretion.

Similar to the *in vivo* results, CE dose-dependently stimulated insulin release from HIT-T15 cells. CE effectively enhanced insulin secretion in high glucose (16.7 mM) containing KRB buffer as well as low-glucose (2.8, 11.1 mM) and glucose-free media.

Glucose stimulates insulin secretion by triggering and amplifying signals in pancreatic β cells. The triggering pathway refers to the K_ATP_ channel-dependent pathway, which is considered to be the major way for glucose stimulated insulin secretion (GSIS). The amplifying pathway partly depends on the activation of PKCs. Although both PMA and CE could promote the secretion of insulin in pancreatic β cells independent of glucose, the activated PKC isoforms in each condition were different. PMA has been shown to activate the cPKCs and nPKCs, but the former plays a critical role in mediating PMA-induced secretion of insulin^12^,^21^. cPKCs were activated by PMA but not CE, suggesting that CE and PMA had different mechanism of action on insulin secretion.

Rottlerin, which selectively inhibits PKC δ, partially attenuated the effect of CE on insulin secretion. Likewise, the insulinotropic effect of CE was not completely suppressed by VE, the DAG inhibitor. DAG functions upstream of PKCs, and its accumulation promotes the activation of cPKCs and nPKCs^22^. Therefore we speculated that CE directly or indirectly activated cPKCs or nPKCs by activating DAG. We found that the enhancement of insulin secretion by CE in HIT-T15 cells required the activation of PKC δ. Other mechanisms are likely involved in the insulinotropic effect of CE, but not cPKC and nPKC activation.

To elucidate the effect of CE on insulin secretion, we analyzed CE-induced activation of different PKC isoforms. The immunoblotting analysis indicated that the insulinotropic targets of CE included not only nPKCs (PKC ε and PKC δ) but also aPKCs (PKC μ and PKC ζ) in HIT-T15 cells. This finding was further supported by immunofluorescence staining of PKC α, δ, μ. In addition, data from siRNA assays of PKC δ, μ, and ζ were also well consistent with the blotting and immunohistochemistry results. Schmitz-Peiffer *et al*. found that the functional ablation of PKC ε selectively enhanced insulin release from diabetic or lipid-pretreated islets^23^, and Mendez *et al*. showed that the translocation of PKC ε from the cytoplasm to the membrane fraction caused a drastic decrease in glucose-stimulated insulin release^24^. Previous studies indicated that GSIS within pancreatic β cells was dependent, at least in part, upon the increase of the translocation of PKC δ, μ, and ζ from cytosol to plasma membrane within the pancreatic β cells^25^,^26^,^27^,^28^. Therefore, membrane translocation performances of PKC ε, PKC δ, PKC μ and PKC ζ in CE-treated cells were in accordance with the previous researches^23^,^24^,^25^,^26^,^27^,^28^.

We presumed that the differential effects of CE effects on different PKC isoforms mainly resulted from their different structures. PKCs are divided into three subfamilies depending on their NH_2_-terminal regulatory domain structure^20^: cPKCs (α, βI, βII and γ) contain a C1 domain and a C2 domain which function as DAG and Ca^2+^ binding motif, respectively; nPKCs (δ, ε, η and θ) have twin C1 domains and a calcium-coordinated-lacking C2 domain, and they can be activated by DAG; aPKCs (ζ, and τ/λ) contain an atypical C1 domain binding phosphatidylinositol triphosphate (PIP_3_) and a protein-protein interaction PB1 (Phox and Bem 1) domain; besides, all isoforms have a domain for binding phosphatidylserine (PS). PKC μ, also called PKD1, is a special isoform that functions like aPKCs but has a structure similar to that of nPKCs^29^. It was classified as a member of aPKCs in this study. Although the activation sites of PKC μ remain unclear, several studies have demonstrated that it could be activated by DAG or PIP_2_^30^. cPKCs were not activated, suggesting that the C1 and C2 domains did not serve as the binding sites of CE, and nPKCs and PKC ζ were probably activated by binding the new C2 domain or the PB1 region. DAG is a common secondary messenger in pancreatic β cells^31^, and its binding site lies in the C1 domain. Thus, CE may not act on DAG directly. VE as a DAG inhibitor, partly suppressed the insulinotropic effect of CE, and the effect of VE was attributed to its reduction of nPKC activity through lowering the levels of DAG in pancreatic β cells. In addition, it has been shown that translocation of PKC μ required phosphorylation in its activation loop^32^ and that this phosphorylation could be caused by activation of nPKCs, such as PKC ε and/or PKC η^33^. Therefore, the activation of PKC μ by CE in this study may be due to changes in the activity of nPKCs. In consequence, nPKCs and aPKCs could be the major targets responsible for the action of CE in the promoting of insulin secretion in HIT-T15 cells.

Many chemicals can increase insulin secretion both *in vitro* and *in vivo*. Currently, the hypoglycemic sulfonylureas are in use as the major drugs to stimulate insulin secretion in diabetic patients. In this study, we speculated that the amplifying pathway of CE was different from that of sulfonylureas. Previous studies have reported that strong activation of PKCs led to increased insulin secretion in pancreatic β cell *in vitro*^34^,^35^, but that it was unlikely that amplifying pathways could ever be stimulated to such an extent *in vivo*. However, CE treatment led to a significant increase of serum insulin and a strong improvement in glucose tolerance. Therefore, simple activation of nPKCs and aPKCs is not sufficient as a therapeutic strategy. CE may have other drug targets in addition to PKCs, and it is this combination of effects that is responsible for its insulinotropic actions.

## Conclusion

In this study, we found that CE, *C. edulis* extract, is a potential insulin secretagogue with a hypoglycemic effect. CE is an insulinotropic extract that acts by selective activation of nPKCs and aPKCs without activating cPKCs. Its insulinotropic and antidiabetic effects were also observed in the HFHS diet-treated ApoE−/− mice, suggesting that CE should could potentially treat hyperglycemia. Although we focused on the effects of CE on insulin secretion in this paper, further study was needed to evaluate other underlying mechanisms of its anti-diabetes effects.

## Methods

### Reagents

Gliclazide was purchased from Tianjin Hua Jin Pharmaceutical Co. Ltd. (Lot: H10910053, Tianjin, China). Commercial total cholesterol (TC), blood glucose and triglycerides (TG) kits for measurement of plasma lipids levels were purchased from Biosino Bio-technology and Science, Inc. (Beijing, China). RPMI-1640 and fetal bovine serum (FBS) were purchased from Mediatech, Inc. (A Corning Subsidiary, Manassas, USA). Horseradish-peroxidase-conjugated secondary antibody and ECL chemiluminescence detection kit were purchased from Applygen Technologies, Inc. (Beijing, China). Insulin was purchased from Humulin, Eli Lilly and Company (Indianapolis, IN, USA). Primary antibodies against PKC α, δ, ζ, and μ were obtained from Cell Signaling Technology (Danvers, MA, USA). PKC βII and N-cadherin were purchased from Abcam (Cambridge, UK). PKC ε and β-actin were obtained from Santa Cruz Biotecnology Inc.(Santa Cruz, CA). FITC-conjugated anti-rabbit antibody Alexa Fluor 488 was from Thermo Fisher Scientific (Boston, MA, USA). Insulin assay for blood samples were determined by enzyme linked immunosorbent assay (ELISA) assays (Mecordia, Uppsala, Sweden). Insulin assay for cell medium was conducted by the HTRF kit (Cisbio, London, UK). SiRNAs were synthesized by Shanghai GenePharma Co.(Shanghai, China). Vigofect reagent was purchased from Vigorous Biotechnology Beijing Co. (Beijing, China).The commercial kit for cytosolic and plasma membrane separation was obtained from Invent Biotechnologies Inc. (Eden Prairie, MN, USA). PMA (the PKC activator), rottlerin (the PKCδ selective inhibitor), vitamin E (the DAG inhibitor), berberine, and all analytical grade solvents were from Sigma-Aldrich (Milano, Italy).

### Plant material, extraction, and characterization of the extract

The whole plants of *C. edulis* were collected from wild in Henan Province, China. The plant materials were authenticated by one of authors (Prof. Jun Li) according to their morphological characteristics. The voucher specimens are deposited in Modern Research Center for Traditional Chinese Medicine, Beijing University of Chinese Medicine. The air-dried whole plants of *C. edulis* (200 g) were refluxed with 75% EtOH twice (2 × 2 L, 2 h each). The extracted solutions were combined and filtered, and the filtrate was evaporated to dryness by a rotary evaporator under reduced pressure at 40 °C to yield 46 g of brown powder (CE). For cell culture, the CE solution was prepared in DMSO solution with the concentration of 40 mg/ml. The CE solution was further diluted with cell culture medium during the cell experiments. HPLC analysis was carried out using an Agilent Extend-C18 column (250 mm × 4.6 mm,  μm, Agilent Technologies, Inc., USA) at an oven temperature 35 °C. The mobile phase consisted of acetonitrile (A) and 0.01 M ammonium formate water (B). The elution gradient was as follows: 0–3 min, linear gradient 3.0–5.0% A; 3–7 min, linear gradient 5.0–14.0% A; 7–20 min, linear gradient 14.0–19.0% A; 20–25 min, linear gradient 19.0–21.0% A; 25–30 min, linear gradient 21.0–30.0% A; 30–40 min, linear gradient 30.0–50.0% A. The flow rate was 1 ml/min. The UV wavelength was set at 254 nm. The typical HPLC profile of CE is shown in Figure S1.

### Ethics statement

All experiments were performed according to the protocol approved by the Animal Care Committee, Peking University Health Science Center (LA2015012). The investigation was carried out in accordance with the Guide for the Care and Use of Laboratory Animals published by the US National Institutes of Health (NIH Publication No. 85–23, revised 1996). All surgeries were performed under anesthesia, and all efforts were made to minimize suffering.

### Animals

Male ApoE−/− mice, weighing 18–22 g, were obtained from the Animal Center of Peking University Health Science Center. Mice were maintained in a controlled environment of 24 ± 1 °C and 50 ± 1% humidity with 12 h of light per 24 h period. Food and water were available *ad libitum*. Mice were randomly divided into six groups (n = 9 for each group): the chow diet-treated group (CG), the HFHS diet-treated group (HG), the low, medium, and high-dose CE treatment groups (CE100, CE200, and CE400, respectively), and the gliclazide treatment group (GG). Mice assigned to CE100, CE200, and CE400 groups were orally administrated 100, 200, and 400 mg/kg/d of CE, respectively; those in the GG group received 100 mg/kg/d of gliclazide for 8 successive weeks. All mice, except for those in CG, were fed a HFHS diet (0.2% cholesterol, 15% sucrose and 10% fat added) for 1 week prior to various treatments. The mice were fed the HFHS diet or chow diet throughout the experiment. Body weight and food intake of each mouse was monitored per 2 weeks.

### FBG, TC, TG and serum insulin assays

Mice were fasted overnight and the blood was collected anticoagulated by heparin. FBG, TC and TG levels were estimated using commercial kits^36^. Insulin levels in blood samples were determined by enzyme linked immunosorbent assay (ELISA) assays.

### IPGTT and ITT

Mice underwent IPGTT and ITT before they were sacrificed. Mice were fasted for 6 h, followed by an intraperitoneal injection of glucose (2 g/kg body weight) or insulin (0.75 mIU/g body weight). Blood samples were collected before the injection (time 0) and at 30, 60 and 120 (90 for ITT) min after the injection for glucose measurement.

### Cell culture of HIT-T15 pancreatic β cell line

HIT cells, subclone T-15, was provided by Prof. Y.F. Guan (Peking University Health Science Center, Beijing, China). Cells were cultured in RPMI-1640 medium supplemented with 20% fetal bovine serum, 100 g/ml streptomycin, and 100 units/ml penicillin in an atmosphere of 5% CO_2_ at 37 °C.

### Insulin secretion assay in HIT-T15 cells

HIT-T15 cells were seeded into 96-well plates and cultured for 24 h. Cells were washed two times using Krebs-Ringer bicarbonate [KRB: 115 mM NaCl, 24 mM NaHCO_3_, 5 mM KCl, 1 mM MgSO_4_, 1.2 mM KH_2_PO_4_, 2.5 mM CaCl_2_, 1 g bovine serum albumin (BSA), buffered with 25 mM HEPES, pH 7.4] buffer. After 1 h of preincubation with KRB (no glucose contained), test incubations were performed with KRB containing various concentrations of glucose and CE for another 1 h (for experiments with agonists or inhibitors, cells were preincubated with them for 30 min before the addition of CE). The cell supernatant was harvested, and an insulin assay was conducted using a HTRF kit.

### Western blot analysis

Cytosolic and plasma membrane fractions were separated using a commercial kit.Samples were separated by 12% SDS-PAGE. Proteins were then electrophoretically transferred to a polyvinylidene fluoride membrane. Subsequently, the membranes were blocked and incubated with antibodies against PKC α, δ, ζ, βII, μ, and ε. β-actin and N-cadherin were used as internal control for cytosolic and plasma membrane respectively. Signals were detected with horseradish peroxidase- conjugated substrates^37^.

### Immunofluorescence microscopy

HIT-T15 cells were seeded on glass bottom cell culture dishes, preincubated with KRB for 1 h, and then not treated (as a control) or stimulated with 100 μg/ml CE for 1 h. After rinsing with phosphate buffered saline (PBS), cells were fixed with 4% paraformaldehyde for 20 min and permeabilized by 0.5% Triton X-100 for 10 min in PBS at room temperature. Nonspecific antibody binding was blocked in PBS containing 5% goat serum for 30 min at 37 °C. Thereafter, cells were stained with anti-PKCα, δ, and μ (1:50), respectively, for 1 h at 37 °C. After three washes with PBS, cells were incubated for 1 h at 37 °C with a FITC-conjugated anti-rabbit antibody (1:100). Localizations of different proteins were examined using a confocal laser microscopy.

### Small interfering RNA (siRNA) transfection

Specific siRNAs against PKC δ, μ, and ζ and scrambled siRNA (N.C.) were designed and synthesized by Shanghai GenePharma Co. and transfected to HIT-T15 cells. The sequences are as follows: si-PKC δ, CACCGGTTCAAGGTTTACAACTA CA; si-PKC μ, CAGCCACCTTTGAAGACT TTCAGAT; si-PKC ζ, GAGCCTCCA.

GTAGATGACAAGAATG; Shuffle: 5′-AACTAGAGCCACAACTACC-3′ was used as control. Transfection was performed using Vigofect reagent according to the manufacturer’s instructions. After 48 h, cells were processed for the following experiments.

### Statistical analysis

Data are expressed as means ± standard error of the mean (SEM) and were analyzed using GraphPad Prism 5.0 for statistical significance. A Student *t*-test or a one-way analysis of variance (ANOVA) was conducted for repeated measurements followed by a post hoc test (Newman–Keuls multiple comparison test) when appropriate. *P* < 0.05 was considered to be statistically significant.

## Additional Information

**How to cite this article**: Zheng, J. *et al. Corydalis edulis* Maxim. Promotes Insulin Secretion via the Activation of Protein Kinase Cs (PKCs) in Mice and Pancreatic β Cells. *Sci. Rep.* **7**, 40454; doi: 10.1038/srep40454 (2017).

**Publisher's note:** Springer Nature remains neutral with regard to jurisdictional claims in published maps and institutional affiliations.

## Supplementary Material

Supplementary Dataset 1
