# Supplementary material for: Corydalis edulis Maxim. Promotes Insulin Secretion via the Activation of Protein Kinase Cs (PKCs) in Mice and Pancreatic β Cells
Source: Sci Rep. 2017 Jan 16;7:40454. doi: 10.1038/srep40454 (PMC5238372; doi:10.1038/srep40454)
Supplement: Supplementary Dataset 1 [file srep40454-s1.doc]

***Supplementary data***

***Corydalis edulis* Maxim. Promotes Insulin Secretion via the Activation of Protein Kinase Cs (PKCs) in Mice and Pancreatic β Cells**

**Jiao Zheng^1, #^, Yunfang Zhao ^1, #^, Qixing Lun ^1, 2, #^, YueLin Song^1^, Shepo Shi^1^, Xiaopan Gu ^1, 2^, Bo Pan ^1, 2^, Changhai Qu ^1^, *, Jun Li ^1^, *, Pengfei Tu ^1^, ***

*^1^ Modern Research Center for Traditional Chinese Medicine, Beijing University of Chinese Medicine, Beijing 100029, China*

*^2^ School of Chinese Materia Medica, Beijing University of Chinese Medicine, Beijing 100102, China*

***Correspondence:**

1 Pengfei Tu, Ph.D.

Professor and Director

Modern Research Center for Traditional Chinese Medicine

Beijing University of Chinese Medicine, Beijing 100029, P. R. China

Tel/Fax: (86)-10-82802750

E-mail: pengfeitu@163.com

2 Jun Li, Ph.D.

Associate Professor

Modern Research Center for Traditional Chinese Medicine

Beijing University of Chinese Medicine, Beijing 100029, P. R. China

Tel/Fax: (86)-10-64286350

E-mail: drlj666@163.com

3 Changhai Qu, Ph.D.

Research assistant

Modern Research Center for Traditional Chinese Medicine

Beijing University of Chinese Medicine, Beijing 100029, P. R. China

Tel/Fax: (86)-10-64286350

E-mail: quchanghai@bucm.edu.cn

# These Authors equally contributed to the work

*Co-corresponding Authors

**Figure S1. Representative HPLC chromatograms of *Corydalis edulis* Maxim. extract (CE).** Chromatographic separation was conducted on an Agilent Extend-C18 column (250 mm × 4.6 mm, 5μm, Agilent Technologies, Inc., USA). The mobile phases were composed of acetonitrile (A) and 0.01M ammonium formate in water (B) using the following gradient program: 0–3 min, linear gradient 3.0–5.0% A; 3–7 min, linear gradient 5.0–14.0% A; 7–20 min, linear gradient 14.0–19.0% A; 20–25 min, linear gradient 19.0–21.0% A; 25–30 min, linear gradient 21.0–30.0% A; 30–40 min, linear gradient 30.0–50.0% A. Flow rate: 1 ml/min. The UV wavelength was set at 254 nm.

**Figure S2. Effect of CE on cAMP content in 16.7 mM glucose.** HIT-T15 cells were exposed to 0–200μg/ml *Corydalis edulis* Maxim. extract (CE) in the presence of 16.7 mM glucose in Krebs-Ringer bicarbonate Buffer (KRB) for 1 h at 37°C. The medium was then removed and 0.1M HCl (0.5 ml/well) was added to the cell pellets. Cell lysates were frozen at -80°C until assayed by enzyme linked immunosorbent assay (ELISA) assays (Cayman Chemical, Inc., Ann Arbor, Michigan, USA). Values are the means ± SE from 6 replicates. Ctrl: DMSO-treated group, CE50: 50 μg/ml CE-treated group, CE100: 100 μg/ml CE-treated group, CE200: 200 μg/ml CE-treated group.
